# Responsible Governance of Tribal Public Health Data: Data Sharing Ethics and Common Challenges in the US Public Health System

**DOI:** 10.2196/77249

**Published:** 2025-06-19

**Authors:** Alec J Calac, Luis R Gasca, William H Swain

**Affiliations:** 1 School of Medicine, University of California, San Diego, 9500 Gilman Drive, San Diego, CA, 92093, United States, 1 (858) 534-2230; 2 Mayo Clinic, Rochester, MN, United States

**Keywords:** American Indian, tribal public health, data sovereignty, ethics, information dissemination, Indigenous peoples, public health surveillance, privacy, data sharing, deidentification, data anonymization, public health ethics, data governance

## Abstract

In their viewpoint, Schmit and colleagues thoughtfully discuss tribal public authority, as well as barriers and facilitators to the responsible use of data generated by or collected from members of sovereign American Indian and Alaska Native Nations. Key topics not covered by the authors that warrant discussion include tribal public health workforce development, data systems infrastructure, and federal facilitation of tribal self-governance programs. These additional topics will better contextualize the ethical, legal, and social issues specific to American Indian and Alaska Native public health practice.

## Tribal Public Health Workforce Development

We read the viewpoint by Schmit and colleagues [1] with great interest and commend the authors for their thoughtful discussion of tribal public authority, as well as the responsible use of data related to members of sovereign American Indian and Alaska Native Nations. There is a nationwide 30% vacancy rate for behavioral health staff, physicians, and other important staff that provide health care and public health services in American Indian and Alaska Native communities [2,3]. In 2025, the National Indian Health Board released the findings of their most recent *Public Health in Indian Country Scan* [3], a report funded by the Centers for Disease Control and Prevention (CDC), which assesses the capacity of tribal health and public health organizations to deliver public health services. This triennial publication helps tribal authorities and other interested parties gain insight into tribal public health systems and identify opportunities for policy and program development. The 2025 report details how the integration of tribal health care and public health services—due to limited staffing and available facilities—can often be problematic. This contrasts the distinct resourcing of these services by their local and state partners, including Tribal Epidemiology Centers, as well as how a lack of dedicated funding and regional training pathways for aspiring American Indian and Alaska Native public health professionals from government agencies like the Indian Health Service (IHS) and CDC contributes to chronic workforce shortages [3]. While the CDC offers epidemiologic assistance to tribal health departments through their long-standing Epidemic Intelligence Service, this does not replace the need for targeted funding that allows tribal entities to develop workforce training pathway programs tailored to local priorities, such as education in epidemiologic research methodologies, data ethics, and disease surveillance [4,5].

## Data Systems Infrastructure

Even with limited access to local, state, and federal public health data systems, tribal public health authorities were able to carry out significant actions to protect the health of their communities during the COVID-19 pandemic and protect individuals most at risk of infection while respecting their privacy rights, pursuant to the Health Information Portability and Accountability Act (HIPAA) [6]. The fragmented nature of the public health system, comprised of the IHS, CDC, Tribal Epidemiology Centers, tribal health entities, and local and state public health departments, ultimately works against improving American Indian and Alaska Native public health outcomes because they do not share real-time, HIPAA-compliant data for the purposes of contact tracing, disease surveillance, occupational hazards, and environmental exposures [6]. Current efforts to rectify this do not address the decentralized nature and limited capabilities of the IHS electronic health record system, or the reality that many nontribal entities do not offer clear guidance on granting tribal public authorities access to their systems [7,8]. The consequences of relying on these data systems to inform public health policy and targeted interventions may contribute to the adverse health outcomes and disparate life expectancy of 65.2 years for American Indian and Alaska Native individuals born today—a figure that is comparable to the American life expectancy almost a century ago [9].

## Federal Facilitation of Tribal Self-Governance Programs

Passage of the 1975 US Indian Self-Determination and Education Assistance Act (ISDEAA) ushered in a new era of self-governance and self-determination efforts meant to facilitate greater tribal control of American Indian and Alaska Native–focused programs and services administered by the federal government. However, to this day, the implementation of these efforts in the US Department of Health and Human Services has been legislatively limited to the IHS Office of Tribal Self-Governance (Public Law 102‐573), which excludes the CDC and other operating divisions that also have significant engagement with American Indian and Alaska Native Nations [10,11]. Outcomes attributable to IHS implementation of tribal self-governance procedures include a 54% decrease in the incidence of end-stage renal failure requiring hemodialysis from 1996 to 2013 (Special Diabetes Program for Indians), local development and operation of tribal institutional review boards, and greater regional uptake of the COVID-19 vaccine than the general population at the height of the pandemic [12-15]. If the CDC were authorized by the US Congress to create a similar office, tribal public health authorities would be able to assume full control of management and funding for public health programs and services that would otherwise be provided to them by the CDC, as well as receive agency expertise and resources to do so under Title V of the ISDEAA [10,11].

## Discussion

The issues discussed in this commentary will not be resolved overnight, but there is at least growing interest to share public health data with tribal authorities at all levels of government [1,7]. The responsible sharing of public health data between local, state, tribal, and federal authorities will ultimately help tribes fulfill their social responsibility to their members and, in turn, help improve public health in the greater community. Public health data reported to the federal government by local authorities may not include information about American Indian and Alaska Native individuals living in urban and rural areas not governed by American Indian and Alaska Native Nations, which may limit the utility of these data when tribal public health authorities request HIPAA-compliant data about American Indian and Alaska Native individuals and communities [7]. Timely sharing of these data at all levels of government will ensure that American Indian and Alaska Native Nations can identify and respond to incidents of public health significance. We look forward to this future.

## Abbreviations:

CDC: Centers for Disease Control and Prevention
HIPAA: Health Information Portability and Accountability Act
IHS: Indian Health Service
ISDEAA: Indian Self-Determination and Education Assistance Act

## References

[1] Schmit CD, O’Connell MC, Shewbrooks S (2025). Dying in darkness: deviations from data sharing ethics in the US public health system and the data genocide of American Indian and Alaska Native communities. J Med Internet Res.

[2] Smith W (2025). Written testimony of William Smith Alaska area representative and chairman, National Indian Health Board before the Senate Committee on Indian Affairs “Hearing on Tribal Priorities of the 119th Congress”. United States Senate Commitee on Indian Affairs.

[3] (2025). Issue brief: building a tribal public health workforce. Public Health Indian Country Capacity Scan.

[4] Indigenous data science education. Indigidata.

[5] Dirlikov E, Fechter-Leggett E, Thorne SL (2020). CDC deployments to state, tribal, local, and territorial health departments for COVID-19 emergency public health response - United States, January 21-July 25, 2020. MMWR Morb Mortal Wkly Rep.

[6] Calac AJ, Hoss A (2022). Vaccine passports and Indian country: nothing fast about it. Public Health Rep.

[7] Field C, Price S, Locklear AC (2024). Barriers and opportunities for tribal access to public health data to advance health equity. J Law Med Ethics.

[8] Mays VM, Echo-Hawk A, Cochran SD, Akee R (2022). Data equity in American Indian/Alaska Native populations: respecting sovereign nations’ right to meaningful and usable COVID-19 data. Am J Public Health.

[9] Parker T, Kelley A (2023). American Indian and Alaska Native life expectancy: writing a new narrative. JAMA.

[10] (2022). National Indian Health Board Resolution 22 – 07: support for legislation expanding tribal self-governance in the Department of Health and Human Services. https://www.nihb.org/wp-content/uploads/2024/12/22-07-NIHB-Resolution-Supporting-Expansion-of-Self-Gov-HHS.pdf.

[11] Office of Tribal Self-Governance (2022). Indian Health Service Tribal Self-Governance Program. https://www.ihs.gov/sites/selfgovernance/themes/responsive2017/display_objects/documents/IHS_OTSG_Brochure.pdf.

[12] ASPE Office of Health Policy (2019). The Special Diabetes Program for Indians: estimates of Medicare savings. ASPE Issue Brief.

[13] Kuhn NS, Parker M, Lefthand-Begay C (2020). Indigenous research ethics requirements: an examination of six tribal institutional review board applications and processes in the United States. J Empir Res Hum Res Ethics.

[14] Haroz EE, Kemp CG, O’Keefe VM (2022). Nurturing innovation at the roots: the success of COVID-19 vaccination in American Indian and Alaska Native communities. Am J Public Health.

[15] Harding A, Harper B, Stone D (2012). Conducting research with tribal communities: sovereignty, ethics, and data-sharing issues. Environ Health Perspect.

